# Infectious Diseases Associated with Livestock Production: Mitigating Future Risks

**DOI:** 10.1289/ehp.121-a256

**Published:** 2013-08-01

**Authors:** Carrie Arnold

**Affiliations:** Carrie Arnold is a freelance science writer living in Virginia. Her work has appeared in *Scientific American*, *Discover*, *New Scientist*, *Smithsonian*, and more.

Humans have traveled with their livestock to the ends of the earth, and animal husbandry has transformed the face of the planet. Even as livestock provide food, these same animals have also introduced humans to new diseases. A study in this month’s *EHP* argues that, although it is unclear whether the intensification of livestock production will lead to a higher risk of disease emergence and transmission, some important risk factors are certainly present.^1^ This is especially true in contexts where changes are occurring rapidly, sometimes before regulation can catch up.

“Strong regulations can do a lot to mitigate zoonotic risk,” says first author Marco Liverani, a social scientist at the London School of Hygiene & Tropical Medicine. But first, he says, we need to better understand which agricultural and economic practices promote emerging infections, if we are to identify vulnerabilities and develop effective policies to address them.

For millennia livestock have shared our abodes and our diseases. The Agricultural Revolution led to the development of larger settlements and denser livestock populations. Infectious diseases that only rarely might have struck small numbers of humans in the past could now spread between animals and large numbers of people. The age of epidemics had begun.^2^

A 2005 study by Mark Woolhouse and Sonya Gowtage-Sequeria, infectious disease specialists at the University of Edinburgh, found that 58% of the 1,407 diseases known to infect humans were zoonotic—that is, they had originated in animals. Pathogens that could infect both animals and humans made up an even bigger proportion of emerging infectious diseases: 73%.^3^

“We have very intense contact with livestock. There are plenty of opportunities for the pathogens to cross over,” Woolhouse says. “There is a lot of exchange of diseases between humans and livestock in both directions.”

The continued growth of the human population, combined with growing demand for animal protein, has further intensified livestock production. The danger in this, according to Liverani and colleagues, is that some practices associated with intensified production have the potential to increase the risk of zoonoses.^1^

Take the emergence of Nipah virus, which first appeared in Malaysia in 1999. Epidemiologists revealed that Nipah was a virus that had never been seen in humans, and ultimately traced its origins to a family of large bats known as flying foxes.^4^

As Malaysia’s population increased dramatically in the late twentieth century,^5^ farmers began growing mangoes and raising pigs in recently deforested areas. Nipah is naturally found in the fruit-eating flying foxes, which feasted on the mangoes planted by farmers. The pigs ate the mangoes that had fallen to the ground, including those that were contaminated by Nipah-laden bat saliva. The pigs caught the virus from the bats, and the humans caught the virus from the pigs.^6^

Although zoonotic disease is a potential issue for large industrial farming operations,^7^ it’s also somewhat easier to regulate these operations, given their resources and commercial status, Liverani says. Backyard farmers like the ones in Malaysia might be exposed to fewer animals, but they tend to have longer, closer contact with the animals that they do have.

**Figure:**
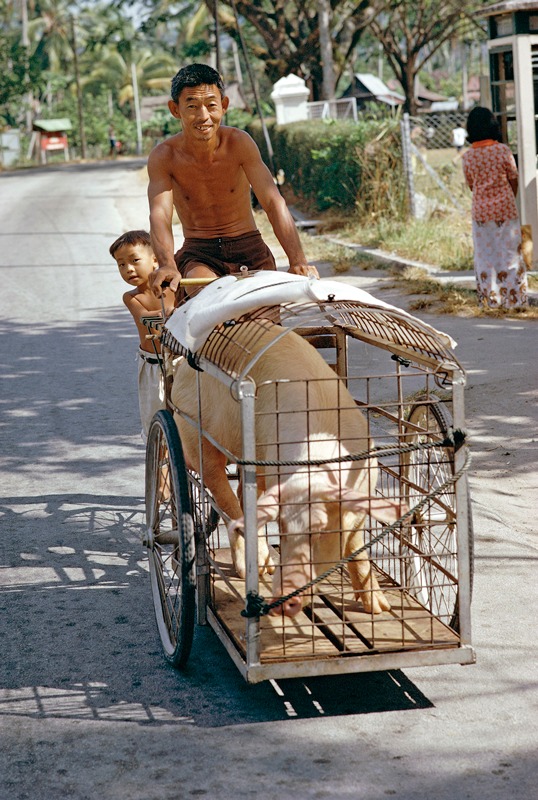
Close contact between humans and livestock can facilitate the exchange of diseases in both directions. © Claire Leimbach

According to Liverani and colleagues, the greatest risk may occur when industrial livestock operations exist alongside small family farms and wildlife populations. Liverani says contacts between livestock and wild animals can result in new pathogens entering intensive production units, where the high concentration of animals can facilitate the amplification and transmission of disease.

Although the dangers of zoonoses are real, Ro McFarlane, a veterinary epidemiologist at the Australian National University in Canberra, cautions that these diseases are only one aspect of the interconnected disease risks that exist between humans, livestock, and the environment. “The intensification of food industries has also given rise to diseases that affect food production,” she says, citing another example of this interconnection. “Many of these are not zoonotic, but the effect on human health through food security and livelihoods can also be catastrophic.”

McFarlane says the strength of the work by Liverani et al. is that it incorporates socioeconomic and political as well as pathogen- and farm-level risk factors into the discussion about how to manage zoonotic risk from intensive livestock industries. “This is the news,” she says—“we are making progress in doing ‘new science.’”
